# Range-wide genetic analysis of *Dermacentor variabilis* and its *Francisella*-like endosymbionts demonstrates phylogeographic concordance between both taxa

**DOI:** 10.1186/s13071-018-2886-5

**Published:** 2018-05-18

**Authors:** Emily L. Kaufman, Nathan E. Stone, Glen A. Scoles, Crystal M. Hepp, Joseph D. Busch, David M. Wagner

**Affiliations:** 1 Pathogen and Microbiome Institute, Northern Arizona University, PO Box 4073, Flagstaff, AZ 86011 USA; 2 USDA, ARS, Animal Disease Research Unit, 3003 ADBF, Washington State University, Pullman, WA 99164 USA; 3 School of Informatics, Computing, and Cyber Systems, Northern Arizona University, PO Box 5693, Flagstaff, AZ 86011 USA

**Keywords:** *Coxiella burnetii*, Cytochrome *c* oxidase subunit 1 (*cox*1), *Dermacentor variabilis*, *Francisella*-like endosymbionts, Mitochondrial phylogeography, *Rickettsia* spp., Ticks, Tick pathogens

## Abstract

**Background:**

The American dog tick, *Dermacentor variabilis*, is an important vector of pathogens to humans, wildlife and domestic animals in North America. Although this tick species is widely distributed in the USA and Canada, knowledge of its range-wide phylogeographic patterns remains incomplete.

**Methods:**

We carried out a phylogenetic analysis of *D. variabilis* using samples collected from 26 USA states and five Canadian provinces. Tick samples (*n* = 1053 in total) originated from two main sources: existing archives (2000–2011), and new collections made from 2012 to 2013. We sequenced a 691 bp fragment of the *cox*1 gene from a subset (*n* = 332) of geographically diverse *D. variabilis*. DNA extracted from individual ticks (*n* = 1053) was also screened for a *Francisella*-like endosymbiont, using a targeted *16S* rRNA sequencing approach, and important pathogens (*Rickettsia* spp. and *Coxiella burnetii*), using species-specific quantitative PCR assays.

**Results:**

Maximum parsimony analysis of *cox*1 sequences revealed two major groups within *D. variabilis* with distinct geographical distributions: one from the eastern USA/Canada (Group 1) and one from the west coast states of the USA (California and Washington; Group 2). However, genetic subdivisions within both of these two major groups were weak to moderate and not tightly correlated with geography. We found molecular signatures consistent with *Francisella*-like endosymbionts in 257 of the DNA extracts from the 1053 individual ticks, as well as *Rickettsia* spp. and *Coxiella burnetii* in a small number of ticks (*n* = 29 and 2, respectively). Phylogenetic patterns for *Francisella*-like endosymbionts, constructed using sequence data from the bacterial *16S* rRNA locus, were similar to those for *D. variabilis*, with two major groups that had a nearly perfect one-to-one correlation with the two major groups within *D. variabilis*.

**Conclusions:**

Our findings reveal a distinct phylogenetic split between the two major *D. variabilis* populations. However, high levels of genetic mixture among widely separated geographical localities occur within each of these two major groups. Furthermore, our phylogenetic analyses provide evidence of long-term tick-symbiont co-evolution. This work has implications for understanding the dispersal and evolutionary ecology of *D. variabilis* and associated vector-borne diseases.

**Electronic supplementary material:**

The online version of this article (10.1186/s13071-018-2886-5) contains supplementary material, which is available to authorized users.

## Background

The American dog tick (*Dermacentor variabilis*) is the most widely distributed native three-host tick in North America and is an important vector of pathogens to humans, wildlife, and domestic animals across the continent [1, 2]. It plays a critical role in the natural maintenance and spread of multiple tick-borne pathogens, such as Spotted Fever group *Rickettsia* spp. (causing Rocky Mountain spotted fever and other spotted fever group rickettsioses), *Francisella tularensis* (causing tularemia), *Coxiella burnetii* (causing Q-fever), and *Anaplasma* spp. (causing anaplasmosis) [3]. *Dermacentor variabilis* is abundant in the eastern USA and southeastern Canada, with a more restricted western population that occurs in the Pacific coast states of the USA; additional disjunct populations have also become established in restricted localities in the intermountain USA and Canada (Alberta, British Columbia, Idaho and Montana) [2, 4–7]. Overall, regional climatic and geographical differences (e.g. temperature and elevation) appear to be the prevailing factors that affect the distributional limits of *D. variabilis* and it has been hypothesized that these factors, along with associated physiological limitations of the tick, could result in genetic divergence of populations [8]. Interestingly, a westward expansion of *D. variabilis* has been documented in recent decades, highlighting the potential for range expansion due to climatic shifts across this region [6, 7, 9].

In the tick gut, endosymbionts and other non-pathogenic microbes significantly outnumber pathogenic organisms [10]. The nature of the relationship between ticks and their symbionts remains understudied and, therefore, poorly understood. Non-pathogenic endosymbiotic bacteria are described from *D. variabilis* and the abundance and composition of this microbiome can vary considerably among populations, even in those that are separated by relatively short geographical distances [5, 11]. The presence of these tick-associated symbionts has been shown to affect the acquisition of some pathogenic bacterial species [12] and, therefore, influence pathogen distributional patterns. A recent study in a very closely related species, *D. andersoni*, demonstrated that there was a negative correlation between the presence of the rickettsial symbiont *R. bellii* and the level to which the tick-borne pathogen *A. marginale* will replicate [13]. Furthermore, the “Rickettsial Exclusion Hypothesis” described the prevention of transovarial transmission of *R. rickettsii* when non-pathogenic endosymbiotic rickettsiae were prevalent [14, 15]; additional studies have experimentally confirmed this rickettsial interference phenomenon with other spotted fever group rickettsiae [16, 17]. The possibility that symbionts might influence competence for tick-borne pathogens has led to an increased interest in the study of endosymbiont prevalence and distribution [18–20].

Genetic analyses of *D. variabilis* are important for understanding its natural history as a host and vector, and to reconstruct its evolution and dispersal mechanisms. This important disease vector boasts an extensive and expanding range, which emphasizes the need for comprehensive phylogeographic studies to better understand its distributional patterns and characterize the relationship between these ticks and their microbiota. Multiple population genetic studies of *D. variabilis* have identified important phylogeographic trends for this species [5, 7, 21–23] and the bacterial pathogens that they vector; however, the breadth of these studies has been limited by geographical scope (1 to 3 states or provinces).

Here, we address this gap in knowledge with a phylogeographic study of range-wide samples using mitochondrial sequencing. The mitochondrial *16S* rRNA and cytochrome *c* oxidase subunit 1 (*cox*1) genes have been used for phylogenetic and population analyses of many tick species in the past [24–31] and have been shown to be phylogenetically informative in discerning relationships from the subfamily level down to that of population. The aims of this study were to: (i) determine if *D. variabilis* exhibits broad population structure throughout its range using sequence data from a large number of individuals and populations representing the known species distribution, and (ii) examine whether there is an association between the presence and population structure of *Francisella-*like endosymbionts and the patterns observed within *D. variabilis*. We also screened all *D. variabilis* samples (*n* = 1053) for *Rickettsia* spp. and *Coxiella burnetii* to better assess the relationship between these two infectious agents and this important arthropod vector.

## Methods

### Sample collection and DNA extraction

To assess phylogeographic patterns of *Dermacentor variabilis* ticks throughout the known geographical range of this species, we obtained individual ticks from two separate sources: (i) existing archives maintained by collaborators (2000–2011), and (ii) new collections made from 2012–2013. For the new collections we provided a sampling kit to collaborators, which included a data sheet for recording sample information, such as host, date of collection, and geographical location. Donated ticks were identified using a morphological dichotomous key [1] and stored at -80 °C until further processing.

Genomic DNA was extracted from 1053 individual whole ticks using the 96-well DNeasy® Tissue Kit (Qiagen, Valencia, CA, USA) modified for use with the QIAvac vacuum filtration system. To begin, individual whole ticks (adult ticks or nymphs) were placed into deep-wells (1.2 ml) of a 96-microtube plate (Qiagen) along with 12 Zirconia beads (Catalog # 7361-002000, 2 mm diameter, Glen Mills, Inc., Clifton, NJ, USA). Lysis solution was prepared and added to the wells with the following protocol modifications: 360 μl Buffer ATL, 40 μl Proteinase K, 100 μl Antifoam-A (Sigma, St. Louis, MO, USA), for a total of 500 μl per well. Each 8-well column was then sealed using round-capped collection microtube caps (Catalog # 19566, Qiagen, Valencia, CA, USA) and placed in a secondary containment heat-sealed 9 × 12 inch Kapak bag (Kapak, Minneapolis, MN, USA) for biosafety purposes. The sealed extraction plates were then placed in a Geno/Grinder 2000 mill homogenizer (SPEX CertiPrep, Metuchen, NJ, USA), which was operated at 2000 strokes/min for 10 min [32]. Following bead-milling, plates were incubated overnight at 56 °C and then subjected to 10 min of additional bead milling the following morning. The remainder of the protocol was carried out in a QIAvac filtration system according to the manufacturer’s guidelines, with a final elution volume of 120 μl (60 μl, twice).

### Mitochondrial *cox*1 sequencing

Many of our sampling locations were represented by an abundance of ticks (*n* > 30 per individual collection) so, to avoid sampling bias, we chose to investigate phylogeographic structure in the mitochondrial *cox*1 gene using a subset (*n* = 332) of our 1053 *D. variabilis* samples from the USA and Canada. To accomplish this, no more than 30 ticks were included from any one sampling location. We designed *cox*1 primers specific to *D. variabilis* that amplified a 793 bp fragment of this gene [COI-F2 (5'-CTT AAT TTT CGG CAG TTG AGC A-3') and COI-R1 (5'-GTT CTT TTT TTC CTG TGG AAA AAC-3')]. PCRs were carried out in 20 μl volumes, using 2 μl of extracted genomic DNA (diluted to 4 ng/μl) as template and the following reagents (given in final concentrations): 1× PCR buffer, 2.5 mM MgCl_2_, 0.2 mM dNTPs, 0.16 U/μl Platinum® *Taq* polymerase (Invitrogen, Carlsbad, CA, USA), and 0.4 μM of each primer. PCRs were thermocycled according to following conditions: 95 °C for 10 min to release the polymerase antibody, followed by 40 cycles of 94 °C for 60 s, 58 °C for 30 s, and 72 °C for 30 s. PCR products were then treated with ExoSAP-IT (Affymetrix, Santa Clara, CA, USA) using 1 μl of ExoSAP-IT per 5 μl of PCR product under the following conditions: 37 °C for 15 min, followed by 80 °C for 15 min. Treated products were then diluted in the range of 1:2 to 1:20 depending on amplicon intensity (as determined by agarose gel electrophoresis) and sequenced in both directions using BigDye® Terminator v3.1 Ready Reaction Mix (Applied Biosystems, Foster City, CA, USA) with the same forward and reverse primers from the initial PCR. We used 10 μl volumes for sequencing reactions containing the following reagents (given in final volumes): 3 μl of 5× sequencing buffer, 1 μl BigDye® Terminator v3.1 Ready Reaction Mix, 1 μl of a 10 μM primer stock, and 5 μl diluted PCR product. The following thermocycling conditions were used: 96 °C for 20 s, followed by 30 cycles of 96 °C for 10 s, 50 °C for 5 s, and 60 °C for 4 min. An ethanol precipitation technique was used to clean and precipitate the DNA pellet, and Sanger sequencing was carried out using an AB3130xl® automated genetic analyzer (Life Technologies, Grand Island, NY, USA). Sequence chromatograms were edited manually in Sequencher 5.0 (Gene Codes, Ann Arbor, MI, USA); after trimming poor quality sequences from each end we were able to use 691 bp of the *cox*1 fragment (from 793 bp in the PCR amplicon). Single nucleotide polymorphisms (SNPs) were identified in MEGA version 6 [33] and a maximum parsimony analysis was employed to construct a phylogenetic tree. The tree was rooted with a *D. reticulatus cox*1 sequence (GenBank: AF132829.1) and a bootstrap analysis of 1000 replicates was used to estimate the robustness of nodes on the tree; bootstrap values were only reported when found to be 50% or greater. To assess the relative amount of homoplasy, the consistency index (CI) and retention index (RI) were calculated following parsimony analysis in MEGA version 6 [33]. The consensus tree was exported into FigTree v1.4.2 (http://tree.bio.ed.ac.uk/software/figtree/), and then into Adobe Illustrator CS4 (Adobe Systems Incorporated, San Jose, CA, USA) for annotation purposes.

We examined the relationship between genetic distance (generated from the 332 *cox*1 sequences) and geographical distance in *D. variabilis* (based upon collection locations) by calculating Spearman’s correlation coefficient using the RELATE function in PRIMER-E software version 6 (Plymouth Marine Laboratory, Plymouth, UK). Analyses were carried out for three datasets: (i) the *D. variabilis* population as a whole (*n* = 332); (ii) individual *D. variabilis* (*n* = 300) assigned to Group 1 in the *cox*1 phylogeny; and (iii) individual *D. variabilis* (*n* = 32) assigned to Group 2 in the *cox*1 phylogeny. We tested the null hypothesis of no relationship between the genetic and geographical distance matrices (ρ = 0) by generating a simulated distribution of randomly sampled data pairs (one from the geographical distance matrix and one from the genetic distance matrix) taken from 10,000 permutations. Additionally, we calculated both within- and between-group mean genetic distances using a maximum composite likelihood function in MEGA version 6 [33].

### Mitochondrial *16S* rRNA sequencing

To assess phylogenetic congruence between two commonly used molecular markers and ensure that conclusions drawn from the *cox*1 gene were supported by an additional mitochondrial locus, we also investigated phylogeographic structure among a small subset (*n* = 32) of our *D. variabilis* samples from the USA and Canada by sequencing a 453 bp fragment of the tick mitochondrial *16S* rRNA gene using primers *16S*+1 (5'-CCG GTC TGA ACT CAG ATC AAG T-3') and *16S*-1 (5'-CTG CTC AAT GAT TTT TTA AAT TGC TGT GG-3') [25]. PCR conditions were identical to the *cox*1 gene, as described above, except an annealing temperature of 58 °C for 60 s was used. Amplicons were visualized, cleaned, and sequenced as described above for *cox*1, but using the primers *16S*+1 and *16S*-1. Sequence editing, SNP identification, phylogenetic analysis, and figure processing were also conducted as described above for the *cox*1 gene, using a *D. andersoni* sample (GenBank: AY375431) as the outgroup.

### *Francisella-*like endosymbiont and pathogen screening

To understand the relationship between *D. variabilis* and specific bacterial species, we screened all samples (*n* = 1053) for the presence of *Francisella*-like endosymbionts using Sanger sequencing, and additionally, several pathogens using species-specific real-time qPCR assays. First, we targeted the bacterial *16S* rRNA locus of *Francisella*-like endosymbionts using conventional PCR with primers developed specifically for these endosymbionts [20, 34, 35]. Because endosymbiont abundance within a tick can be low, leading to false negative results, all individuals were screened in duplicate to increase the likelihood of detection. Primers F11 (5'-TAC CAG TTG GAA ACG ACT GT-3'; *Escherichia coli 16S* positions 150–169) [34] and 1227R (5'-CCA TTG TAG CAC GTG T-3'; *E. coli 16S* positions 1227–1242) [35] were used to generate a ~1093 bp amplicon (1090 bp in *Francisella* due to two separate deletions). A standard 20 μl PCR was performed with 2 μl (diluted to 20 ng/μl) of the extracted genomic DNA as template and the following reagents (given in final concentrations): 1× PCR buffer, 2.5 mM MgCl_2_, 0.2 mM dNTPs, 0.16 U/μl Platinum® *Taq* polymerase, and 0.4 μM of each primer. PCRs were thermocycled using the following conditions: 95 °C for 10 min to release the polymerase antibody, followed by 40 cycles of 94 °C for 60 s, 56°C for 30 s, and 72 °C for 1 min 30 s. In the absence of *Francisella*-like endosymbionts, this *16S* assay occasionally amplified other bacterial species. For this reason, all *16S* positive PCR products were then sequenced to confirm the identity and presence of *Francisella*-like endosymbionts. PCR visualization, cleaning, and sequencing methods were the same as described above for the *cox*1 gene but used the primers F11 and 1227R. Sequences were trimmed on each end to a final length of 725 bp (*E. coli 16S* positions 253–972). Sequence editing, SNP identification, phylogenetic analysis, and figure processing was conducted as described above, with a *F. tularensis* subsp. *holarctica* sample (GenBank: MG834502) as the outgroup.

All *D. variabilis* DNA extracts (*n =* 1053) were also screened for the presence of *Rickettsia* species and *C. burnetii* DNA using species-specific qPCR assays. To facilitate rapid analysis of all ticks (*n* = 1,053), preliminary screening for *Rickettsia* species was carried out using the Pan-*Rickettsia* real-time PCR assay [36], a TaqMan® qPCR assay that targets the 23S rRNA gene using “PanR8” oligonucleotides. This real time assay is able to detect a number of *Rickettsia* species, including *R. rickettsii*, *R. prowazekii* and *R. typhi*, all of which have been reported in North America and cause human infections [37, 38]. Reactions were carried out in 10 μl volumes using 1 μl of extracted genomic DNA (pre-diluted to 4 ng/μl) as template and the following reagents (given in final concentrations): 2× AB TaqMan® Universal master mix (Life Technologies, Grand Island, NY, USA), 1 μM of both the forward and reverse primer, and 0.04 μM probe. Plates were analyzed using an ABI 7900HT Sequence Detection System (Life Technologies, Grand Island, NY, USA); two positive controls (mean C_T_ 19.8) along with 6 NTCs (no amplification) were included with each run. To obtain species level identification of all *Rickettsia* positive samples from the previous qPCR (C_T_ < 40, *n* = 29), a 530 bp segment of the *OmpA* gene was amplified and sequenced. Primers Rr190.70p (5'-ATG GCG AAT ATT TCT CCA AAA-3') and Rr190.602n (5'-AGT GCA GCA TTC GCT CCC CCT-3') [39] were used in a 25 μl PCR, performed with 3 μl of the extracted genomic DNA (pre-diluted to 20 ng/μl) and the following reagents (given in final concentrations): 1× 5 PRIME MasterMix (5 PRIME, Hilden, Germany) and 0.4 μM of each primer. PCRs were thermocycled according to the following conditions: 94 °C for 3 min to release the polymerase antibody, followed by 32 cycles of 94 °C for 45 s, 56 °C for 1 min, and 72 °C for 1 min 30 s. PCR products were visualized, cleaned, and sequenced as previously described, but using the primers Rr190.70p and Rr190.602n. Sequence editing, SNP identification, phylogenetic analysis, and figure processing was conducted as described previously. Additionally, we screened for the presence of *C. burnetii* among our *D. variabilis* individuals using the IS1111 TaqMan® real-time qPCR genotyping assay, following the conditions set forth by Loftis et al. 2006 [40]; six positive controls (mean C_T_ 9.2) along with 12 NTCs (no amplification) were included with each run.

## Results

### Mitochondrial *cox*1 sequencing

**Fig. 1:**
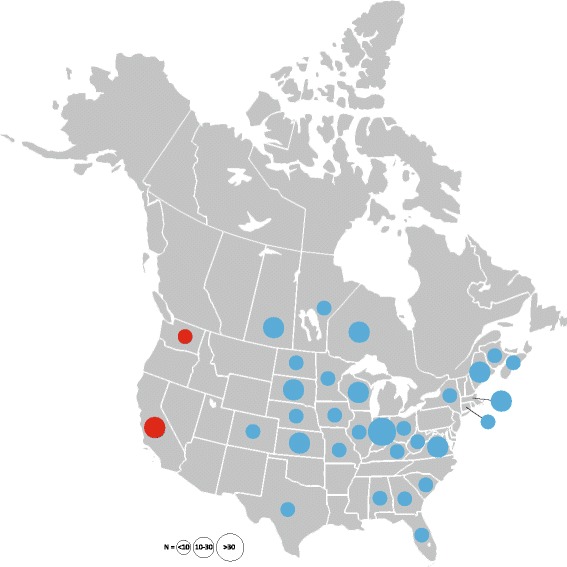
Sampling distribution of 332 *D. variabilis* ticks sequenced at the mitochondrial *cox*1 gene. Circles are placed at the geographical center of each state or province and the relative area of each circle is proportional to sample size. Colors indicate membership to one of the two major phylogenetic groups (blue, Group 1; red, Group 2) within the entire *D. variabilis* population

**Fig. 2:**
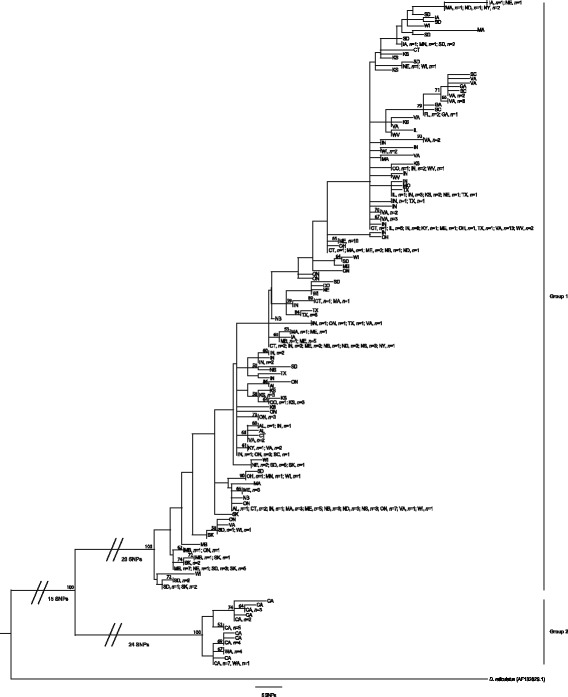
Phylogenetic relationships among 332 geographically diverse *D. variabilis* ticks from the USA and Canada. A maximum parsimony consensus of three equally parsimonious mitochondrial *cox*1 trees is given, illustrating a deep phylogenetic split within the greater *D. variabilis* population. *D. reticulatus* serves as the outgroup species

We identified two major groups within *D. variabilis* (bootstrap support = 100%) that separate according to distinct geographical distributions (Fig. 1 and Additional file 1: Table S1): one occurs in the eastern USA/Canada (Group 1) and the other is restricted to west coast states in the USA (CA, WA; Group 2). This result is in agreement with the major pattern observed in a previous study that investigated the mitochondrial *16S* gene in a limited number of *D. variabilis* collections from the western USA and Canada [5]. We found a large amount of genetic diversity within the 691 bp *cox*1 fragment: Group 1 was comprised of 118 unique mitochondrial haplotypes, whereas Group 2 had just 12 haplotypes. In total, 110 nucleotide positions were variable (i.e. had at least one SNP): 71 positions were parsimony informative, and 39 positions were not parsimony informative. The vast majority of SNPs in this *cox*1 segment were synonymous; only 10 nucleotide substitutions were nonsynonymous and each was limited to an individual tick and, therefore, parsimony uninformative. More than 60% of the informative synonymous SNPs (*n* = 44) separated Group 1 from Group 2. The mean genetic distance between the two major *D. variabilis* clades was 0.055. In contrast, the mean genetic distance was an order of magnitude lower within samples from Group 1 and Group 2 (0.005 and 0.003, respectively). The consensus tree acquired from three equally parsimonious trees is presented in Fig. 2, with CI of 0.577 and RI of 0.966. This tree is a condensed version of the full maximum parsimony tree with identical genotypes collapsed together; it is labeled with information on collection locations (states and/or provinces) and sample size from each location. An additional expanded version of this tree, including identification and other information on each individual, is presented in Additional file 2: Figure S1.

Several states/provinces (especially KS, ME, ON, SD, VA) had a mixture of dissimilar haplotypes that were spread across the entire phylogeny. The number of unique haplotypes per state or province ranged from two in WA to 20 in IN. In addition, the single most common haplotype (found in 30/332 ticks, or 9%) was widely distributed; it occurred in 11 states and provinces from Alabama to New Brunswick and was relatively basal compared to most other haplotypes. However, a few smaller lineages appeared to display more restricted distributions that were only observed farther north (ND, SD, NE, KS, IA, MN, WI, NY, MA) or restricted to the southeastern USA (VA, SC, GA, FL).

Within the two major groups, we observed very few genetic subdivisions that were associated with geography. The correlation between genetic and geographical distance within the eastern ticks (Group 1) was moderate (ρ = 0.179, *P* < 0.0001), and samples from Group 2 did not reveal a significant correlation (*P* > 0.05). The most robust relationship between geographical distance and genetic distance was observed when all *D. variabilis* samples were used (ρ = 0.453, *P* < 0.0001, which reflects the large genetic and geographical separation between ticks from Group 1 and Group 2.

### Mitochondrial *16S* rRNA sequencing

A similar *D. variabilis* phylogeny, including the major division between Group 1 and Group 2, was obtained using a 453 bp sequence of the *16S* rRNA gene from 32 geographically diverse *D. variabilis* individuals (Additional file 1: Table S1). The consensus maximum parsimony tree acquired from five equally parsimonious trees is shown in Additional file 3: Figure S2, with CI of 0.900 and RI of 0.989. However, the *16S* RNA gene yielded less phylogenetic resolution than that of the *cox*1 gene; this was determined by comparing the percentage of variable nucleotide sites, and additionally, the number of haplotypes represented within each gene fragment [*16S* = 18 SNPs (3.97%) within a 453 bp segment; resulting in 8 haplotypes *vs cox*1 = 52 SNPs (7.53%) within a 691 bp segment; resulting in 15 haplotypes] using 25 samples that were analyzed for both genes (Additional file 1: Table S1). As a result, no additional individuals were sequenced at this locus.

### *Francisella-*like endosymbiont and pathogen screening

**Fig. 3:**
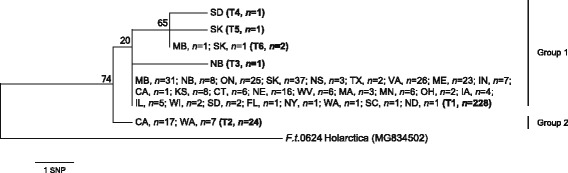
Phylogenetic relationships among 257 geographically diverse *Francisella-*like endosymbiont sequences from the USA and Canada. A maximum parsimony bacterial *16S* rRNA tree is given, illustrating a distinct genetic split within the endosymbiont population, which mirrors that of the tick host

The main genetic discontinuity that distinguishes *D. variabilis* in the western USA (Group 2) from all other locations (Group 1) is also reflected in the *Francisella*-like endosymbionts of these ticks (Fig. 3). Of the 1053 *D. variabilis* individuals screened, 257 ticks produced robust *16S* sequences that were highly similar to the *Francisella*-like endosymbionts known to be associated with several *Dermacentor* species [20]. It is important to note that, due to the low abundance of endosymbiont DNA in our samples, our assay likely did not detect all positive individuals. The endosymbiont *16S* sequences from Group 1 ticks were primarily a single sequence type labelled “T1” (*n* = 228). We also found five *16S* variants in endosymbionts from eastern ticks that differed by 1–2 SNPs; types T3 through T6 were 97–98% identical. All Group 2 ticks from CA and WA shared a distinct endosymbiont *16S* type (T2), with just two exceptions (CA, *n* = 1; WA, *n* = 1) that had the T1 signature (Fig. 3). One of these ticks (D.v.0411 CA) was sequenced at the *cox*1 locus and was assigned to *D. variabilis* Group 2. This could indicate that T1 endosymbionts have been translocated to the western lineage of *D. variabilis* at some point. A single SNP (corresponding to *E. coli 16S* position 258) separates the T2 (A nucleotide) from T1 (G nucleotide) endosymbiont lineages and occurs at the end of a hairpin stem in *16S* region 2. All endosymbionts shared a 2-bp insertion (immediately downstream of *E. coli 16S* position 478) in a side loop located in variable region 3; this insertion appears to be unique to the endosymbiont sequences. The other endosymbiont *16S* SNPs in groups T3 through T6 were rare and found only in ticks from the northern distribution of *D. variabilis* (i.e. 5 ticks from SD, MB, NB and SK). The consensus maximum parsimony tree for the 257 *Francisella*-like endosymbionts is shown in Fig. 3, with CI of 1.000 and RI of 1.000; an expanded tree provides specific information for individual samples (Additional file 4: Figure S3). Interestingly, we detected relatively high levels of genetic differentiation among endosymbionts found in *D. variabilis* compared to that within other *Francisella* species (Additional file 5: Figure S4).

Bacterial pathogens were detected among our 1053 *D. variabilis* samples but in low frequency. *Rickettsia* spp. were detected in 29 tick samples (C_T_ < 40; Additional file 1: Table S1). These 29 *Rickettsia-*positive samples produced robust *OmpA* sequences that were highly similar to *Rickettsia* species known to be associated with several *Dermacentor* ticks in North America [41, 42]. Most notably, amplicons consistent with *R. montanensis* were detected in 20 individual ticks (1.9% prevalence among all individuals screened), amplicons consistent with *R. peacockii* in 8 ticks (0.8% prevalence), and one amplicon was consistent with a *Rickettsia* endosymbiont from *Ixodes scapularis* (WI tick)*.* Most of the positive ticks (27 out of 29) were sampled in central and eastern Canada from 2000 to 2010. A maximum parsimony tree for rickettsial sequences (Additional file 6: Figure S5) provides location information for individual ticks. We note that, due to the low abundance of *Rickettsia* DNA in the samples, our assay likely did not detect all positive individuals. Using the IS1111 assay, low levels of *C. burnetii* were detected in two tick samples: D.v.0451 IN (C_T_ 42.1) and D.v.0239 NB (C_T_ 37.5) (Additional file 1: Table S1).

## Discussion

Despite the extensive geographical distribution of *D. variabilis* in North America, and the critical role that these ticks play in the transmission of pathogenic viruses and bacteria to humans and animals, little work has been done to characterize the diversity within this species across its entire range. Past studies have demonstrated the utility of the mitochondrial *16S* rRNA locus in detecting genetic variation among *D. variabilis* populations [5], but few other markers have been used for phylogeographic studies of the American dog tick. In this study, we generated sequence data for two mitochondrial markers (*cox*1 and *16S*) to improve the understanding of genetic structure across the range of this species and to correlate observed patterns with components of its microbiome. Our analysis of both of these markers suggests that the *cox*1 locus provided the appropriate genetic resolution for addressing our study questions. Here we present the first range-wide patterns of genetic structure and distribution in *D. variabilis* ticks across North America while also establishing a correlation in phylogenetic patterns between *D. variabilis* ticks and one of their bacterial symbionts.

We found strong support for the previously noted genetic divergence between eastern (Group 1) and western (Group 2) populations of *D. variabilis* [5, 22]. The relatively large number of SNPs that separate these two groups (as compared to the limited diversity within these groups) suggests that geographical isolation has played a role in shaping their genetic divergence. There are two prevailing hypotheses that could explain why these two major groups have distinct geographical distributions. First, it is possible that *D. variabilis* was recently introduced to the west coast by means of a founder event. This could have been the result of a single event or a small number of dispersal events in which a rare mtDNA type from an eastern population became established in the west and persisted until the present, with no subsequent gene flow back to the east. *Dermacentor variabilis* does use large mammalian hosts with the ability to travel long distances over a short period of time (e.g. native canids or domestic dogs transported by humans) and these hosts could have been the source for the initial establishment of *D. variabilis* on the west coast, bridging the continental divide that separates these two populations. This scenario would require gravid female ticks to remain attached to the host for the duration of travel, and then detach in an environment suitable for oviposition and survival of the offspring. But a founder event like this would be unlikely because: (i) the duration of adult attachment to the host is probably too short for long-distance movement on a wild host; and (ii) detachment of a gravid female in a suitable habitat is not likely across great portions of the dry intermountain region of the western USA. In a second scenario, it is possible that *D. variabilis* individuals on the west coast represent a relict population, whose original range was once more widespread and possibly contiguous with the eastern population during a past geologic epoch. Under this model, we suggest that changes in the availability of refugia during previous glacial episodes could explain the current geographical isolation of these groups [43]. Subsequent habitat discontinuity would then serve to enforce their long-term genetic isolation. A separate study focusing on the North American distribution of the *Ixodes ricinus* complex [27] found 16 SNPs (4.7%) within a 338 bp segment of the mitochondrial *16S* rRNA gene. These SNPs separated northern and southeastern clades and were estimated to represent 35,000 years of evolution. In the current study, we found a similar ratio of SNP variation (6.4%) in the *cox*1 gene that separated the two major *D. variabilis* lineages. Although the *cox*1 appears to mutate at a faster rate than *16S* in *D. variabilis*, the similar ratio of mitochondrial SNPs in *D. variabilis* and *I. ricinus* complex may indicate a long-time genetic separation of the two *D. variabilis* groups, lending support for the glacial refugia scenario.

Within the two main phylogenetic groups, the weak genetic differentiation in *D. variabilis* populations at a regional scale suggests high levels of genetic mixture, which is probably a consequence of host movements. Ticks are highly vulnerable in the environment when off-host, so their survival and dispersal are intrinsically linked to host behavior and movement [44], which can also influence phylogeographic patterns. *Dermacentor variabilis* ticks are commonly found on companion animals, such as domesticated dogs, and the potential for anthropogenic influence on host/vector dispersal is quite high. In this study, *D. variabilis* demonstrated little genetic structure among neighboring populations but strong genetic differentiation among distantly separated populations, indicating high levels of regional dispersal, possibly *via* domesticated dogs (Fig. 2 and Additional file 2: Figure S1); this observation has also been noted in previous studies [7, 44, 45].

The east-west population divergence observed in both endosymbionts and their tick hosts is suggestive of a long-term co-evolutionary relationship in which the endosymbiont populations have diverged in parallel with their vector hosts. Indeed, the amount of genetic differentiation observed within the *Francisella*-like endosymbionts from this study is much greater than that observed within other *Francisella* species, such as *F. tularensis*, *F. piscicida*, and *F. noatunensis* (Additional file 5: Figure S4), suggesting that the endosymbionts have been coevolving with *D. variabilis* for a very long time. Genetically-isolated tick populations can serve as “islands” where genetic drift can lead to differentiation of both the ticks and the pathogens and symbionts that they harbor. For example, local adaptation in *D. variabilis* populations has been used to explain a notable regional difference in its ability (and inability) to transmit *A. marginale* [22]. Similarly, the microbiome of *D. andersoni* differs from region to region, which may have an impact on competence for *A. marginale* in this species as well [11, 13, 46]. Because endosymbionts may alter the acquisition of pathogenic species, and a geographical correlation appears to exist among ticks, pathogens, and endosymbionts, these findings highlight that a better understanding of vector-microbe relationship may have implications for managing vector-borne diseases.

## Conclusions

Ticks are a highly successful and diverse group of parasites, and studies of their natural history, behavior, and genetic/geographical structure are critically important to improve our understanding of their efficiency as vectors, and for implementing effective strategies for controlling ticks and the pathogens they transmit. Phylogenetic analyses allow us to approximate the magnitude, direction, and timing of genetic dispersal, investigate species microevolution, and make inferences about the future distribution of various tick species. So far, studies of dispersal, colonization, and population genetics demonstrate that tick populations continue to harbor considerable diversity in their behavior and genetic structure, and species-specific biological information is needed to accurately deepen our understanding of ticks and the pathogens they transmit. The present study provides the first data concerning an in-depth phylogeographic analysis of *D. variabilis* and the *Francisella*-like endosymbionts associated with this tick species across its entire range and provides evidence of long-term tick/symbiont co-evolution. Using *cox*1 mtDNA sequencing, we found two distinct genetic clades that suggest present-day populations may have been established during a recent glacial period.

## Additional files

Additional file 1: **Table S1.** *Dermacentor variabilis* (*n* = 1053) were obtained from 31 states and provinces. We included a subset (*n* = 491), which represents all *D. variabilis* that produced data for ≥ 1 analysis (*cox*1 phylogeny, *16S* phylogeny, *Francisella*-like endosymbiont detection and *16S* phylogeny, *Rickettsia* spp. detection and *OmpA* phylogeny, and *Coxiella burnetii* detection). A subset were sequenced at *cox*1 (*cox*1^*D.v.*^, *n* = 332), and/or *16S* (*16S*^*D.v.*^, *n* = 32). We also include ticks that were positive and/or sequenced at three species targets: *Francisella*-like endosymbiont *16S* (*16S*^E^, *n* = 257), *Rickettsia OmpA* ([R], *n* = 29), and *C. burnetii* ([C], *n* = 2). (XLSX 67 kb)

Additional file 2: **Figure S1.** Expanded maximum parsimony phylogeny of 332 *Dermacentor variabilis* from the USA and Canada based on the *cox*1 gene. A consensus of three equally parsimonious trees is given. Stars indicate samples for which mitochondrial *16S* sequences were also generated. (EPS 1788 kb)

Additional file 3: **Figure S2.** Expanded maximum parsimony phylogeny of 32 *Dermacentor variabilis* from the USA based on the *16S* rRNA gene. A consensus of five equally parsimonious trees is given illustrating a phylogenetic split within the greater *D. variabilis* population, which is congruent with the *cox*1 phylogeny. (EPS 2121 kb)

Additional file 4: **Figure S3.** Maximum parsimony phylogeny of 257 *Francisella-*like endosymbiont *16S* rRNA gene sequences illustrates a distinct split within the endosymbionts, which mirrors the tick host. (EPS 4528 kb)

Additional file 5: **Figure S4.** Maximum parsimony phylogenetic analysis among six *Francisella* species and the *Francisella*-like endosymbionts from this study based on the *16S* rRNA gene illustrates a high level of diversity among endosymbionts when compared to closely related *Francisella* species. (EPS 1279 kb)

Additional file 6: **Figure S5**. Maximum parsimony phylogenetic analysis of the *OmpA* gene in *Rickettsia* spp. isolated from *Dermacentor variabilis*. This phylogeny represents the three *Rickettsia* species and one *Rickettsia-*like endosymbiont detected during this study. (EPS 1202 kb)

## Abbreviations

CI: Consistency index
*cox*1: Cytochrome *c* oxidase subunit 1 gene
NTC: Non-template control
PCR: Polymerase chain reaction
qPCR: Quantitative PCR
RI: Retention index
SNP: Single nucleotide polymorphism
